# Toxic shock syndrome with a cytokine storm caused by *Staphylococcus simulans*: a case report

**DOI:** 10.1186/s12879-020-05731-y

**Published:** 2021-01-06

**Authors:** Ken Goda, Tsuneaki Kenzaka, Masahiko Hoshijima, Akihiro Yachie, Hozuka Akita

**Affiliations:** 1 Department of Internal Medicine, Hyogo Prefectural Tamba Medical Center, 2002-7 Iso, Hikami-cho, Tamba, 669-3495 Japan; 2 Division of Community Medicine and Career Development, Kobe University Graduate School of Medicine, 2-1-5 Arata-cho, Hyogo-ku, Kobe, 652-0032 Japan; 3 Department of Pediatrics, Kanazawa University, 13-1, Takaramachi, Kanazawa, 920-8641 Japan

**Keywords:** Coagulase-negative staphylococcus, Cytokine storm, *Staphylococcus simulans*, Toxic shock syndrome

## Abstract

**Background:**

Exotoxins secreted from *Staphylococcus aureus* or *Streptococcus pyogenes* act as superantigens that induce systemic release of inflammatory cytokines and are a common cause of toxic shock syndrome (TSS). However, little is known about TSS caused by coagulase-negative staphylococci (CoNS) and the underlying mechanisms. Here, we present a rare case of TSS caused by *Staphylococcus simulans* (*S. simulans*).

**Case presentation:**

We report the case of a 75-year-old woman who developed pneumococcal pneumonia and bacteremia from *S. simulans* following an influenza infection. The patient met the clinical criteria for probable TSS, and her symptoms included fever of 39.5 °C, diffuse macular erythroderma, conjunctival congestion, vomiting, diarrhea, liver dysfunction, and disorientation. Therefore, the following treatment was initiated for bacterial pneumonia complicating influenza A with suspected TSS: meropenem (1 g every 8 h), vancomycin (1 g every 12 h), and clindamycin (600 mg every 8 h). Blood cultures taken on the day after admission were positive for CoNS, whereas sputum and pharyngeal cultures grew *Streptococcus pneumoniae* (Geckler group 4) and methicillin-sensitive *S. aureus*, respectively. However, exotoxins thought to cause TSS, such as TSS toxin-1 and various enterotoxins, were not detected. The patient’s therapy was switched to cefazolin (2 g every 8 h) and clindamycin (600 mg every 8 h) for 14 days based on microbiologic test results. She developed desquamation of the fingers on hospital day 8 and was diagnosed with TSS. Conventional exotoxins, such as TSST-1, and *S. aureus* enterotoxins were not detected in culture samples. The serum levels of inflammatory cytokines, such as neopterin and IL-6, were high. CD8+ T cells were activated in peripheral blood. Vβ2+ population activation, which is characteristic for TSST-1, was not observed in the Vβ usage of CD8+ T cells in T cell receptor Vβ repertoire distribution analysis.

**Conclusions:**

We present a case of *S. simulans*-induced TSS. Taken together, we speculate that no specific exotoxins are involved in the induction of TSS in this patient. A likely mechanism is uncontrolled cytokine release (i.e., cytokine storm) induced by non-specific immune reactions against CoNS proliferation.

## Background

Toxic shock syndrome (TSS) caused by a *Staphylococcus aureus* infection is a relatively rare complication of influenza [1]. The diagnostic criteria defined by the Council of State and Territorial Epidemiologists of the United States includes fever (38.9 °C or higher), diffuse erythematous dermatosis, desquamation (1–2 weeks after the onset of rash), hypotension (systolic blood pressure < 90 mmHg), and multi-system involvement (three or more organ systems) [2]. Exoproteins associated with *S. aureus* act as superantigens. They non-selectively bind to MHC class II antigens on antigen-presenting cells, activate a large number of T cells through T-cell receptor interaction, and then induce the secretion of cytokines [3]. TSST-1 and a series of enterotoxins have been reported as such superantigens [4].

However, little is known about TSS caused by coagulase-negative staphylococci (CoNS) [5, 6]. It remains to be determined how TSS is induced by the CoNS infection. An immune reaction to the proliferation of CoNS organisms that causes cytokine activation has been proposed as an underlying mechanism [5].

Here, we report the case of a patient with TSS caused by *Staphylococcus simulans* (*S. simulans*)*,* a CoNS species, after pneumococcal pneumonia associated with influenza.

## Case presentation

**Fig. 1:**
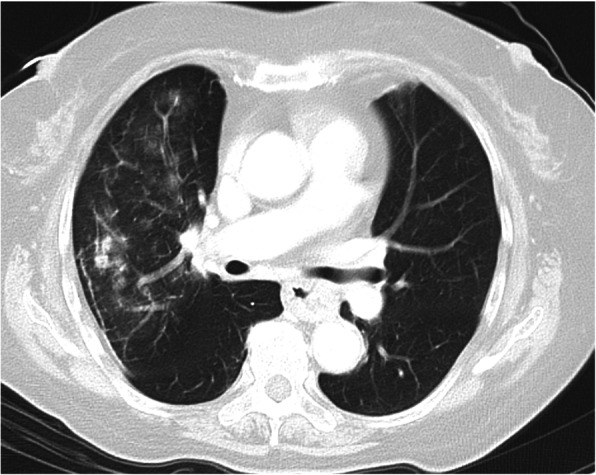
Chest computed tomography on admission

A 75-year-old woman, who was being treated with prednisolone 5 mg/day for polymyalgia rheumatica, presented with anorexia and general malaise for 3 days and then developed vomiting and diarrhea. She experienced difficulty moving her body, and her consciousness deteriorated. The patient was brought to our hospital on an ambulance. The consciousness level on admission, body temperature, blood pressure, pulse rate, respiratory rate, and peripheral oxygen saturation were E3V4M6, 39.5 °C, 153/88 mmHg, 124 beats/minute, 25 breaths/minute, and 90% (with oxygen mask 6 L/min), respectively. Her bulbar conjunctiva was congested on both sides, and coarse crackles were heard in the right lung field. Reticular dermatosis was found on both of her lower legs, and erythematous dermatosis was observed on the lower side of the back. Laboratory blood tests on admission showed elevated inflammatory markers (white blood cell count and C-reactive protein), creatine phosphokinase, and liver aminotransferase. Further, a rapid influenza diagnostic test was positive for influenza A (Table 1). Chest computed tomography (CT) showed diffuse airspace opacification in the right middle lobe (Fig. 1). Head CT demonstrated a slight fluid collection in the right upper sinus suggesting sinusitis.

**Table 1:** Laboratory data at admission

Parameter	Recorded value	Reference value
White blood cell count	5.37 × 10^3^/μL	4.50–7.50 × 10^3^/μL
Neutrophils	89.6%	42–74%
Lymphocytes	5.6%	18–50%
Hemoglobin	12.6 g/dL	11.3–15.2 g/dL
Hematocrit	37.6%	36–45%
Platelets	179 × 10^3^/μL	130–350 × 10^3^/μL
C-reactive protein	5.0 mg/dL	≤0.60 mg/dL
Total protein	6.5 g/dL	6.9–8.4 g/dL
Albumin	3.3 g/dL	3.9–5.1 g/dL
Aspartate aminotransferase	134 U/L	11–30 U/L
Alanine aminotransferase	156 U/L	4–30 U/L
Lactate dehydrogenase	323 U/L	109–216 U/L
Creatine phosphokinase	404 U/L	40–150 U/L
Blood nitrogen urea	20.2 mg/dL	8–20 mg/dL
Creatinine	0.58 mg/dL	0.63–1.03 mg/dL
Sodium	136 mEq/L	136–148 mEq/L
Potassium	3.2 mEq/L	3.6–5.0 mEq/L
Glucose	166 mg/dL	70–109 mg/dL
Rapid influenza test	Positive for type A

Her clinical signs and symptoms fulfilled three out of five clinical criteria of TSS with a fever of 38.9 °C or higher, diffuse erythroderma, and multisystem involvement (conjunctival congestion, gastrointestinal symptoms of vomiting and diarrhea, hepatic dysfunction, and disorientation). At this time point, she had not developed desquamation yet and had no hypotension. Therefore, the patient was clinically diagnosed with influenza with bacterial pneumonia and potential TSS, and her treatment was started with meropenem (1 g every 8 h), vancomycin (1 g every 12 h, therapeutic drug monitoring: ≥15 μg/mL), and clindamycin (600 mg every 8 h).

The next day, *S. simulans*, a CoNS strain, was detected in both sets of blood cultures. Furthermore, pneumococci and methicillin-sensitive *Staphylococcus aureus* (MSSA) were identified in sputum cultures (Geckler 4 group) and pharyngeal cultures (small number of colonies), respectively. Exotoxins, such as TSST-1, and staphylococcal enterotoxins (type A, B, C, and D), which are known to cause TSS, were not detected in culture samples of MSSA and *S. simulans*. We used the reversed passive latex agglutination method to evaluate the exotoxins and enterotoxins; the minimum detection sensitivity of sensitized latex using this method was found to be 1–2 ng/mL. We changed the antimicrobial therapy to cefazolin (1 g q8h) and clindamycin (600 mg q8h), based on microbiologic test results, and this therapy was administered for 14 days. Finger desquamation appeared on the 8th day of hospitalization, fulfilling four out of five clinical criteria for TSS. Therefore, we diagnosed the case as probable TSS.

**Fig. 2:**
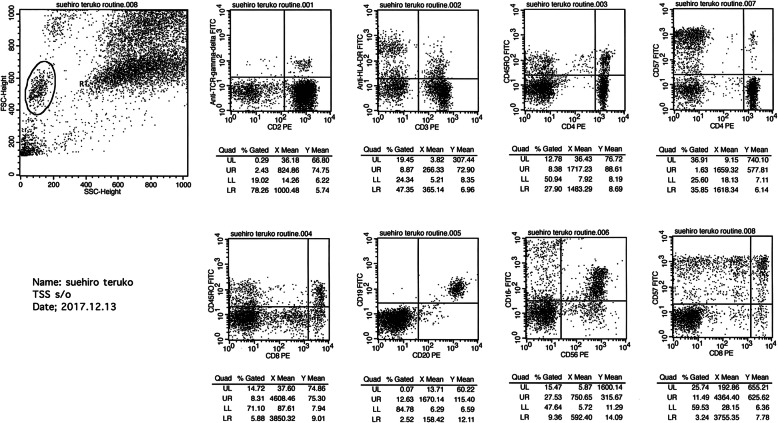
Lymphocyte subpopulation distribution in peripheral blood

Serum levels of inflammatory cytokines were high: neopterin 62 mmol/L (reference range, ≤5 mmol/L) and IL-6 38 pg/mL (reference range, ≤5 pg/mL). Lymphocyte subpopulation distribution was then analyzed in peripheral blood (Fig. 2), showing prominent CD57 expression in CD8+ T cells, suggesting strong activation of this T lymphocyte subset.

**Fig. 3:**
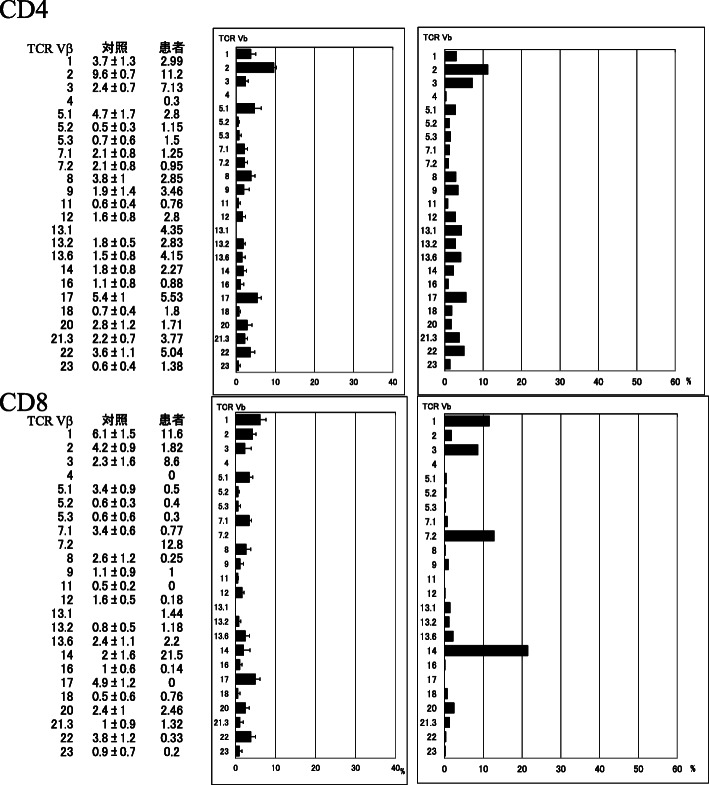
T cell receptor (TCR) Vβ repertoire distribution analysis in peripheral blood

Furthermore, T cell receptor (TCR) Vβ repertoire distribution analysis was carried out (Fig. 3). A specific subset of CD8+ T cells, including Vβ7.2 and Vβ14 cells, was increased, indicating the oligoclonal activation of CD8+ T cells likely due to antigen exposure. However, the distribution of CD4+ T cell TCR Vβ repertoire was normal. The activation of Vβ2+ population, which is characteristic for TSST-1, was not observed.

## Discussion and conclusions

The occurrence of TSS by CoNS is extremely rare, and only a few cases have been reported [5, 6]. The current patient was diagnosed with probable TSS (non-streptococcal), as she fulfilled the five diagnostic criteria proposed by the Council of State and Territorial Epidemiologists (USA) and Centers for Disease Control and Prevention (US). Furthermore, the underlying mechanism not only involves superantigens but also interactions among bacterial cell wall components and monocytes, leading to the release of cytokine mediators [5].

Generally, TSS is caused by *S. aureus* infection. In the common forms of TSS, exotoxins, such as TSST-1, and a series of staphylococcal enterotoxins non-selectively act as superantigens on MHC class II antigens on antigen-presenting cells and TCR on T-cells, causing the activation of a large number of T cells and systemic secretion of cytokines [3]. Known exotoxins causing TSS were undetectable in the current case using the reversed passive latex agglutination method.

*S. simulans* is a residential type of CoNS in animals, such as cattle, sheep, and goats, and it is sometimes pathogenic to humans [7]. There have been reports of bacteremia, infective endocarditis, and cellulitis [8]. It has been reported that blood cultures become positive within 30 h from culture initiation, when CoNS is the causative organism [9]. Contrarily, 46.7% of *S. aureus* are exotoxin-producing strains, and only 26.7% of CoNS strains produce exotoxins [10]. Whether exotoxins secreted by CoNSs are capable of inducing TSS remains unknown.

In the acute phase of common staphylococcal TSS, Vβ2-positive T cells increase more than several times compared to the normal values stimulated mainly by the exotoxin TSST-1 [11]. We observed relatively selective increases in Vβ7.2 and Vβ14-positive CD8+ T cells in the current patient. This type of TCR Vβ selectivity has not been linked to known superantigens [12] and may be a unique response to some specific antigens. In fact, there has been no report on the analysis of the TCR Vβ repertoire in a patient with TSS caused by CoNS.

In the current case, the levels of inflammatory cytokines, including neopterin and IL-6, were high, suggesting a strong activation of the immune system known as cytokine storm [13]. It remains to be determined how TSS is induced by CoNS infection. The distinct pattern of TCR Vβ selectivity in the current case coordinates well with the negative results of TSST-1 and enterotoxin tests. A proposed mechanism has been that an immune reaction to the proliferation of CoNS organisms causes cytokine activation [5]. This warrants further studies investigating TCR Vβ selectivity in TSS associated with CoNS.

In conclusion, we present a case of *S. simulans*-induced TSS. We speculate that no specific exotoxins were involved in the induction of TSS in this patient. A likely mechanism is the uncontrolled cytokine release (i.e., cytokine storm) induced by non-specific immune reactions against CoNS proliferation.

## Data Availability

Data sharing is not applicable to this article as no datasets were generated or analyzed during the current study.

## Abbreviations

CoNS: Coagulase-negative staphylococcus
CT: Computed tomography
MSSA: Methicillin-sensitive *Staphylococcus aureus*
*S. simulans*: *Staphylococcus simulans*
TCR: T cell receptor
TSS: Toxic shock syndrome
